# Inflammatory and Molecular Pathways in Heart Failure—Ischemia, HFpEF and Transthyretin Cardiac Amyloidosis

**DOI:** 10.3390/ijms20092322

**Published:** 2019-05-10

**Authors:** Diana Michels da Silva, Harald Langer, Tobias Graf

**Affiliations:** 1 Department of Cardiology, Angiology and Intensive Care, Medicine Medical Clinic II, University Heart Center Lübeck, 23562 Lübeck, Germany; diana.michelsdasilva@uksh.de (D.M.d.S.); harald.langer@uksh.de (H.L.); 2 German Center for Cardiovascular Research (DZHK), Partner Site Hamburg/Kiel/Lübeck, 23562 Lübeck, Germany

**Keywords:** cardiac inflammation, HFrEF, HFpEF, TTR amyloidosis

## Abstract

Elevated pro-inflammatory biomarkers and cytokines are associated with morbidity and mortality in heart failure (HF). Preclinical and clinical studies have shown multiple inflammatory mechanisms causing cardiac remodeling, dysfunction and chronic failure. Therapeutics in trials targeting the immune response in heart failure and its effects did not result in evident benefits regarding clinical endpoints and mortality. This review elaborates pathways of immune cytokines in pathogenesis and worsening of heart failure in clinical and cellular settings. Besides the well-known mechanisms of immune activation and inflammation in atherosclerosis causing ischemic cardiomyopathy or myocarditis, attention is focused on other mechanisms leading to heart failure such as transthyretin (TTR) amyloidosis or heart failure with preserved ejection fraction. The knowledge of the pathogenesis in heart failure and amyloidosis on a molecular and cellular level might help to highlight new disease defining biomarkers and to lead the way to new therapeutic targets.

## 1. Introduction

Heart failure (HF) is a complex syndrome characterized by the inability of the heart to uphold sufficient blood flow due to systolic or diastolic dysfunction. Roughly 26 million people worldwide are affected [1], while the prevalence in industrialized nations has increased to more than 10% in the elderly >70 years of age [2]. The prognosis of patients with chronic HF is marked by repeated hospitalizations and mortality, which is about 50% within 5 years of initial diagnosis [1]. Therefore, a better understanding of the underlying pathophysiological mechanisms of HF is essential in order to develop and improve therapeutic measures and thereby reduce mortality. This is no easy feat, seeing as etiology of HF is diverse. Most common causes of cardiac dysfunction are ischemia, mechanical stress and volume overload. However, other conditions such as valvular heart disease, myocardial infarction (MI), autoimmune or storage diseases deserve utmost attention. Currently, much focus is on studying the pathophysiology of ischemic HF and HFpEF (HF with preserved ejection fraction). The former is characteristically associated with reduced systolic function as a consequence of ischemic cell death and inadequate elimination of toxic metabolic degradation products. HFpEF on the other hand results from continuous pressure and volume overload and is characterized by diastolic dysfunction due to increased fibrosis and reduced ventricular compliance [3]. Extensive experimental and clinical research has shown that the pathogenesis of chronic HF is mediated by a complex inflammatory response that initially facilitates tissue reparation, but when persistent promotes cardiac adverse remodeling and dysfunction [4,5,6]. These findings are substantiated by a positive correlation between inflammatory mediators and left ventricular dysfunction [7,8,9]. To date, the therapy of HF concentrates on ameliorating workload and contractility via manipulation of the neurohormonal axis and has proven effective in HFrEF (HF with reduced ejection fraction). Unfortunately, no pharmacological therapy has proven to be similarly effective in HFpEF [10]. This is a worrisome revelation, seeing as HFpEF accounts for roughly 50% of all HF cases and exhibits growing prevalence [11]. Hence, identifying pathognomonic mechanisms in HFpEF that are open to modulation could be momentous for any future therapy. In this context, transthyretin amyloidosis (ATTR) is increasingly garnering recognition as an underdiagnosed cause of diastolic dysfunction. In HFpEF patients ≥60 years, the prevalence of relevant transthyretin amyloid deposition reached 13% [12], while the prevalence in patients ≥75 years increased to 32% [13]. With demographic developments in mind, targeted therapies for ATTR cardiomyopathy are needed and fortunately already in development and under investigation.

## 2. Pathways in Ischemic Heart Failure 

Ischemic HF is characterized by three phases: an acute inflammatory phase, a reparative phase (day 4–14 after MI) and chronic inflammation (>14 days after MI). Early inflammation and reparative mechanisms are triggered by ischemia and necrotic cardiomyocytes. Regeneration of the myocardium, comprising removal of dead or irreparable tissue and formation of scar tissue, is initiated by the infiltration of neutrophils followed by monocytes and macrophages, which are released from splenic reservoirs mediated by angiotensin II (ATII) [14] or are produced in the bone marrow through IL-1β-signaling [15,16]. In mice, two types of monocytes differentiated by surface expression markers CD14 and CD16 are predominant in early inflammation: Ly6C^high^ (human homolog: CD14^++^CD16^−^) and Ly6C^low^ (CD14^+^CD16^++^) monocytes [17]. The latter are recruited through interaction between monocytic receptors and chemokines (e.g., CCR2 and CCL2) [18] as well as cardiac endothelial cells and cell adhesion molecules on monocytes including intracellular adhesion molecule 1 (ICAM1), vascular cell adhesion protein 1 (VCAM1) and E-/P-selectin [19]. Ly6C^high^ monocytes secrete several proinflammatory cytokines such as TNF-α, IL-1β and proteolytic enzymes like matrix metalloproteinases (MMPs) and recruit inflammatory macrophages (M1) tasked with digesting necrotic cells and damaged extracellular matrix (ECM) [17]. As with monocytes, chemotaxis of macrophages is mediated by chemokines with affinity to either M1 and/or M2 macrophages [20]. The effect of TNF-α is dependent on whether TNF receptor 1 (TNFR1) or 2 (TNFR2) is bound. While TNFR1 knock-out mice showed improved remodeling with heightened cardiac contractility and reduced NF-κB activation after MI, TNFR2 knock-out mice exhibited exaggerated remodeling with increased fibrosis accompanied by left ventricular dilatation and dysfunction [21,22]. Likewise, NF-κB signaling has contrary effects depending on time of activation and surrounding cell environment and can either enhance hypertrophy, contribute to cytoprotection from ischemia or act cytotoxic by prolonging inflammation [23]. 

The transition from acute inflammation to tissue reparation is driven by successively reduced pro-inflammatory cytokine production in M1 macrophages during phagocytosis. Simultaneously, secretion of anti-inflammatory and profibrotic cytokines interleukin-10 (IL-10) and transforming growth factor beta (TGF-β) as well as pro-angiogenic factors are increased [24]. This change in cytokine profile is promoted by neutrophils, that help recruit monocytes/macrophages and polarize macrophages towards this reparative (“alternative”) phenotype termed M2 [25,26]. Depletion of neutrophils in mice subjected to MI leads to decline in cardiac function, increased fibrosis and progressive HF [25]. Thus, despite promoting tissue injury when continuously recruited, presence of neutrophils during inflammation is also vital for cardiac repair. M2 macrophages can be further differentiated and either coordinate adaptive immune response (M2a and M2c) or suppress inflammation (M2b) and facilitate healing [27,28]. During the healing phase, Ly6C^high^ monocytes are relieved by Ly6C^low^ monocytes responsible for triggering tissue regeneration by promoting myofibroblast formation (contractile α-smooth muscle actin expressing fibroblast) and collagen production through TGF-β secretion and removal of pro-inflammatory IL-1β, inhibiting myofibroblast conversion, inducing angiogenesis through vascular endothelial growth factor (VEGF) secretion, influencing composition of ECM by regulating MMPs and activating the adaptive immune response by antigen-presentation to lymphocytes [27,29,30]. 

During the inflammatory phase of MI, reactive oxygen species (ROS) and IL-1 stimulate generation of pro-inflammatory fibroblasts that secrete cytokines and chemokines [31]. This is achieved by IL-1 mediated inhibition of α-smooth muscle actin expression, which in turn delays conversion to myofibroblasts [31]. During transition to the healing phase, fibroblasts differentiate to myofibroblasts and produce collagen fibers in order to form scar tissue and uphold myocardial integrity [32]. This process is termed reparative or replacement fibrosis. In the event of sustained or recurring inflammation, reactive fibrosis impairs cardiac contractility and function due to imbalance between fibroblasts and viable cardiomyocytes [32]. This form of maladaptive fibrosis is also a key pathology in HFpEF. Numerous factors coordinate post-MI myofibroblast trans-differentiation, the best-characterized being the cytokine TGF-β. TGF-β is secreted by leukocytes, thrombocytes and fibroblasts in the infarct area and is activated in response to ROS, activation of proteases and mechanical strain [29,33].

It is pivotal for the preservation of repaired cardiac structure and function that inflammatory responses, which initially contribute in a positive way, do not persist. In fact, studies in animals and HF patients have shown that sustained inflammation is a cornerstone of adverse cardiac remodeling and chronic HF and is marked by an abundance of M1 macrophages, lymphocytes and other pro-inflammatory mediators [34,35,36]. Macrophages were found to migrate into the remote (non-infarcted) myocardium, while numbers in scar tissue fell [17]. Inhibition of leukocyte recruitment to the infarcted and remote myocardium using RNAi targeting cell adhesion molecules attenuated adverse cardiac remodeling, thus proving the long-term pro-inflammatory role of monocytes/macrophage in adverse post-MI heart remodeling [17,37]. In line with these findings, elevated blood monocyte counts were shown to predict impaired ejection fraction [38]. Recent studies have illustrated that the cardiac lymphatic system is in part responsible for the removal of immune cells from the myocardium and that vascular endothelial growth factor-C (VEGF-C) therapy optimizes healing in the infarcted heart [39]. Accordingly, genetic deletion of the lymphatic vessel endothelial hyaluronan receptor 1 (LYVE-1) in infarcted mice with subsequent decrease in leukocyte clearance to the mediastinal lymph nodes leads to adverse cardiac remodeling [40].

Fundamental research throughout the years has repeatedly verified the presence of the adaptive immune system in acute inflammation and cardiac remodeling [41,42], but research investigating the roles of T and B lymphocytes, antibodies and dendritic cells in HF is rather sparse. There is increasing evidence that a persistent, pathological low-level activation of anti-cardiac autoimmunity through self-antigen presentation upholds and aggravates adverse cardiac remodeling. Tissue damage and necrosis after MI is followed by the release of cardiac antigens (e.g., α-myosin heavy chain [43] and troponin) that are recognized as danger-associated molecular patterns (DAMPs) and induce local and systemic inflammation [44]. In the course of cardiac reparation, a combination of physiological mechanisms that restrain autoimmune activation and the decline in DAMPs should ultimately terminate the adaptive immune response. During MI however, great amounts of cardiac antigens are released, overpowering restrictive and tolerance mechanisms and effecting a prolonged autoimmune response with persistent tissue damage and release of self-antigens which in turn results in a self-reinforcing cycle of chronic inflammation [45]. Experimental murine models provided evidence that B lymphocytes contribute to adverse cardiac remodeling. In an ATII infusion model, cardiac remodeling was compared between wild type mice and mice either lacking B and or T cells [46]. In B cell depleted mice negative remodeling was less detrimental and when B cells were reconstituted left ventricular hypertrophy and fibrosis were increased. Additionally, in B cell positive mice expression of pro-inflammatory cytokines such as IL-1β, IL-6 and TNF-α as well as immunoglobulin G3 were significantly higher than in B cell depleted mice [46,47]. Furthermore, B lymphocytes act in a pro-inflammatory manner by mobilizing monocytes and modulating the T cell response by acting as antigen presenting cells (APC) [48]. Similar to B lymphocytes, T lymphocytes were shown to advance chronic HF. TAC (transverse aortic constriction) mice models with induced T cell deficiency and T cell receptor alpha knockout mice indicated amelioration of systolic function, prevention of ventricular dilation and reduction of fibrosis [49,50]. These effects were reversed after T lymphocyte reconstitution in the first model. Moreover, CD4^+^ T cells displayed a greater negative impact on cardiac remodeling than CD8^+^ cells [51], whereby the latter may additionally exhibit direct cytotoxic effects [52]. In contrast, T regulatory cells demonstrate a cardioprotective role and attenuate cardiac remodeling, inter alia, through IFN-γ [52,53]. Surprisingly, CD4^+^ T cells also produce IFN-γ next to pro-inflammatory cytokines such as IL-17 in MI, which could be the indication of a dichotomous role [54,55]. Moreover, CD4^+^ knockout mice demonstrated impaired healing in the infarct zone, suggesting that CD4^+^ T lymphocytes may also facilitate cardiac reparation [54]. Dendritic cells (DCs) are responsible for antigen presentation and influence phenotyping of T lymphocytes and may therefore induce an exaggerated effector T cell response in severe inflammation following MI. In patients with dilated cardiomyopathy and myocardial infarction, a decreased population of DCs was associated with worsening of systolic function, impaired reparative fibrosis, increased cardiac rupture and unfavorable short-term outcome [56,57]. Conversely, blockade of T cell co-stimulation with DCs, B lymphocytes and macrophages using abatacept improved cardiac function and delayed disease progression [58]. 

## 3. Pathways in HFpEF

In some parts, mechanisms in development and progression of HFpEF resemble those in ischemic HF. Mechanical stress caused by pressure or volume overload affects the release of ATII, which in turn stimulates mobilization of Ly6C^high^ monocytes from the spleen and bone marrow to the myocardium [59]. What follows is the already detailed cellular and immune cascade including infiltration with M1 macrophages, secretion of pro-inflammatory cytokines and chemokines, differentiation to M2 macrophages and reactive fibrosis stimulated by TGF-β secretion [60,61,62]. With that said, ineffectiveness of current HF pharmacotherapy, especially RAAS-inhibition, in HFpEF seemed incomprehensible [63,64,65], but gave rise to the assumption that HFpEF must be caused by a distinct pathophysiology that differs from ischemic HF. Endomyocardial biopsies and further investigation into signaling pathways revealed that diastolic dysfunction in HFpEF is characterized by the following pathologies: myocardial interstitial fibrosis, cardiomyocyte hypertrophy and stiffness and capillary rarefraction [66,67,68]. The myocardial cyclic guanosine monophosphate (cGMP)-protein kinase G (PKG) signaling pathway plays a pivotal role in the formation of aforementioned structural changes. Physiologically, nitric oxide (NO) and natriuretic peptides activate soluble and particulate guanylate cyclase that in turn generate cGMP [69,70]. Next, cGMP activates PKG which in turn phosphorylates numerous proteins, regulates cytoplasmatic Ca^2+^ homeostasis influencing cardiomyocyte contractility, inhibits hypertrophy and promotes left ventricular relaxation and compliance by phosphorylation of troponin I and titin [69]. Titin is a sarcomeric protein with spring-like properties enabling early diastolic recoil and late diastolic distensibility and exists in two isoforms: the larger and more compliant N2BA isoform and the smaller and stiffer N2B isoform [71,72]. PKG, among other kinases, reduces stiffness of titin via phosphorylation [72,73]. Hence, cardiomyocyte stiffness varies based on dynamic expression of titin isoforms and extent of phosphorylation. In HFpEF, hypophosphorylation is far more present than in HFrEF and results in increased cardiomyocyte stiffness [74,75]. Reasons for hypophosphorylation are reduced myocardial PKG activity and cGMP levels which are both downregulated due to increased microvascular inflammation and oxidative stress [76]. Recurring mechanical stress and systemic inflammation increase the production of ROS in cardiac myocytes and cardiac endothelial cells. ROS in turn binds NO, hence reducing its bioavailability and thereby downregulating NO-cGMP-PKG signaling [77]. Furthermore, increased levels of ROS can directly activate the TGF-β/Smad3 pathway promoting fibrosis [78]. The cardioprotective effects of NO-cGMP-PKG signaling have been exemplified in in recent studies. In rat models of HFpEF stimulation of soluble guanylyl cyclase (sGC), which generates cGMP, by utilization of the NO-independent stimulator BAY 41-8543 showed less cardiac fibrosis, macrophage infiltration and gap junction remodeling as well as improved diastolic function and hemodynamics, and less susceptibility to ventricular arrhythmias [79,80]. Likewise, acute cGMP enhancement with the phosphodiesterase type 5A inhibitor sildenafil and infusion of brain-natriuretic peptide ameliorated LV diastolic distensibility in dogs in part by increased phosphorylation of titin [81]. 

These finding gave way to the hypothesis that comorbidities associated with systemic endothelial inflammation are the driving force behind emergence and progression of HFpEF [82]. Indeed, amongst patients with HFpEF prevalence of comorbidities marked by systemic inflammation and endothelial dysfunction such as obesity, diabetes mellitus type 2, hypertension, metabolic syndrome, atrial fibrillation, pulmonary diseases, renal dysfunction and anemia are high [83]. Some of these comorbidities also present with reduced myocardial capillary density which is promoted by microvascular endothelial inflammation and impairs myocardial perfusion affecting ventricular dysfunction [65,84,85]. Furthermore, microvascular endothelial inflammation stimulates migration of leukocytes and the subsequent inflammatory cascade previously described [82]. This detailed comorbidity-driven, phenotypic heterogeneity makes HFpEF far more complex than initially assumed and must first be fully understood in order to guarantee adequate planning of clinical trials and therapy management [86].

## 4. Novel Therapeutics in Heart Failure—Immunosuppression, Immunomodulation, Regeneration

Discovery of chronic inflammation as a pivotal component of development and progression of HF paved the way for extensive research targeting suppression and modulation of immune responses and regeneration of cardiac tissue. Unfortunately, results have consistently been inconclusive and conflicting, so that as yet not one therapy is fit for routine clinical application [87] (Table 1). Major problems in statistical meta-analysis and ascertainment of therapeutic benefit are heterogeneity of study populations, variation in treatment regime and timely initiation as well as differing endpoints and follow-up periods. Furthermore, clinical trials have concentrated on proving favorable effects foremost in the acute/post-MI phase and less on long-term cardiac development and in chronic HF. Several immunosuppressive or -modulatory drugs are well-established treatments in autoimmune diseases or prevention of transplant rejection and have been repurposed for the treatment of acute inflammation in MI. A detailed listing of all drugs and related clinical trials would go beyond the scope of this review. The most extensively studied therapeutics however shall be discussed in the following. 

Broad immunosuppression has been attempted with corticosteroids, methotrexate (MTX), cyclosporin A (CsA) and intravenous immunoglobulins (IVIg) that inhibit recruitment and activation of the innate and adaptive immune responses via multiple pathways and directly protect cardiomyocytes from cell death due to ischemia/reperfusion injury [116,117,118,119,120]. Meta-analyses of corticosteroid trials and recent singular trials (e.g., COPE-ADHF [88]: dexamethasone followed by prednisolone for 7 days in acute decompensated HF) have shown decreased mortality in HF and cardiac arrest [89,90] patients, but it remains unclear whether this improvement derived from cardioprotective effects. Two representative trials studying MTX—METIS [93] (patients with chronic HF) and TETHYS trial [91] (patients with ST-segment elevation myocardial infarction (STEMI))—found no significant changes in inflammatory and cardiac biomarkers, infarct size, New York Heart Association (NYHA) class, 6-min walk test (6MWT) and mortality, but worsened left ventricular ejection fraction (LVEF) at 3 to 4 months follow-up. The ongoing clinical trial CIRT (Cardiovascular Inflammation Reduction Trial) [92] aims at evaluating the effect of low-dose MTX on atherothrombosis and prevention of adverse cardiovascular events. Completed trials assessing the use of CsA in patients with MI have, in sum, not produced any clinically relevant results, albeit a meta-analysis of five randomized control trials of acute MI patients showed significant reduction in peri-operative myocardial injury and post-operative rise in cardiac troponin T [94]. Due to ambiguous results, no definitive statement on the potential positive effect of IVIg on cardiac function and structure can be made. While Gullestad et al. [96] and McNamara et al. [97] demonstrated up-regulation of anti-inflammatory cytokines and improved LVEF after IVIg infusion in patients with chronic HF and dilated cardiomyopathy at 6 months follow-up respectively, no effect was noted in another trial by Gullestad et al. in patients with acute MI post percutaneous coronary intervention (PCI) undergoing the same treatment regime [95]. 

Seeing as numerous cytokines are involved in cardiac inflammation following ischemia and mechanical stress, research has devoted itself to developing specific agents inhibiting cytokine function, whereby much focus has been dedicated to IL-1, IL6 and TNF-α. Currently, two types of drugs are being tested in regards to IL-1: the IL-1 receptor antagonist anakinra and monoclonal antibodies canakinumab and gevokizumab that neutralize IL-1β. Clinical trials evaluating anakinra have so far delivered contradicting results. The most common finding was down-regulation of systemic inflammation in HF and acute coronary syndrome identified through measurement of C-reactive protein (CRP) and other pro-inflammatory factors [99,100,101]. Improvements in LVEF and diastolic function however were inconsistent, and no study showed reduction in major adverse cardiac events during the short-term follow-up period. A recent trial from van Tassell et al. in HFpEF patients similarly observed favorable trends in CRP and NT-proBNP, but the primary efficacy endpoint—improved cardiorespiratory fitness—was not met [98]. A follow-up trial VCU-ART 3 (NCT01950299) in patients during the acute phase of STEMI is currently underway. Similar to anakinra, canakinumab reduced inflammatory parameters in patients with previous MI and type 2 diabetes with high risk of MI [103,121]. The largest trial to date — CANTOS [121] — applying canakinumab in 10,061 patients with previous MI additionally detected reduced incidence of non-fatal MI, non-fatal stroke and cardiovascular-related death after 2 years follow-up, while a secondary analysis also found improvement of peak oxygen consumption and LVEF (from 38% to 44%) [104]. A recent study saw a dose-dependent reduction in hospitalization in patients with HF and prior MI [122]. A novel IL-1β antibody, gevokizumab, formerly intended for non-infectious uveitis, has so far only been tested in animal models, whereby healthy and diabetic rats showed immediate sustained improvement of ischemia-/reperfusion-induced cardiac and coronary dysfunction [122]. The majority of studies involving TNF-α were based on populations with HF. After smaller studies [123,124] investigating etanercept, a TNF-α receptor antagonist, identified positive effects such as improved LVEF, 6MWT and NYHA class larger randomized, placebo-controlled trials were initiated. The trials RECOVER and RENAISSANCE studied patients with chronic HF with NYHA III and IV receiving etanercept in differing weekly dosage and unfortunately found no clinical or survival benefit after nearly 2 years, bringing on termination of both trials [106]. Joint analysis of both trials (RENEWAL) came to the same conclusion [106]. Equally disappointing were results from the ATTACH trial that treated patients with moderate to severe chronic HF (NYHA III/IV) with infliximab, a monoclonal antibody and TNF inhibitor [107]. Despite lowering levels of inflammatory markers (CRP and IL-6) no significant improvement of clinical status could be determined, while higher doses caused relevant adverse effects.

ROS plays an essential role in early inflammation and perpetuation of tissue damage in ischemic HF and HFpEF and therefore represent promising therapeutic targets. However, data on N-acetylcysteine (NAC), a ROS blocker, have thus far only been acquired from populations with acute MI or STEMI. Results regarding NAC have been promising. The latest study—the NACIAM trial—of STEMI patients undergoing PCI and receiving NAC and nitrate therapy observed a doubling of myocardial salvage and reduction of infarct size by 5.5% compared with placebo along with symptomatic improvement [110]. Cardiac functional parameters however were not significantly changed, though it should be acknowledged that observation only lasted 7 days and therefore no conclusion regarding long-term effects can be drawn. Recently, sGC, an enzyme activated by NO and involved in the cGMP-PKG signaling pathway which benefits cardiac remodeling has become a target for therapy in HFpEF. Vericiguat, a sGC stimulator, was given to patients with HFpEF and improved quality of life, even though NT-proBNP and left atrial volume remained unchanged [111]. 

Due to late recognition of adaptive immunity as a key player in sustained low-level inflammation, studies inhibiting recruitment and activation of effector T and B lymphocytes are limited. CsA was meant to target effector T lymphocytes by inhibiting transcription of cytokines and co-receptors critical for their recruitment and function, but as already detailed above, had no effect on cardiac structure, function or all-cause mortality [94]. In experimental MI models, antibody-mediated depletion of CD20 or B cell activating factor, a factor associated with higher mortality rate in patients with recurrent MI, recruitment of B cells was reduced while cardiac function improved [48]. The currently recruiting RITA-MI trial (NCT03072199) plans to study the anti-CD 20 antibody rituximab in acute MI patients. Another approach that could benefit HF would be to boost the regulatory T cell population that counterbalances ongoing inflammation. Supporting this strategy, are findings regarding adenosine, which is known to increase regulatory T cell numbers. A meta-analysis of 15 randomized, controlled trials in 1736 patients with acute MI undergoing PCI and receiving adenosine reported reduced rates of HF development, though LVEF and mortality were unchanged [112]. The first clinical trial specifically targeting regulatory T lymphocytes is yet to come. 

Several other immunomodulatory drugs have been or are currently under investigation such as IL-6 inhibitors (e.g., tocilizumab [105]), complement 1 and 5 inhibitors (e.g., C1-INH [108] and pexelizumab [109]), mast cell stabilizers (e.g., tranilast [113]), histamine receptor 2 antagonists (e.g., famotidine [114]), CD11/CD18 integrin inhibitors [115], mineralocorticoide receptor antagonists (e.g., eplerenone [125]) and phosphodiesterase inhibitors (e.g., pentoxifylline [126], milrinone [127] and sildenafil [80]). Furthermore, device therapy such as cardiac resynchronization therapy [128] and low-level transcutaneous vagus nerve stimulation [129] have attenuated cardiac remodeling in man and mouse. Lastly, regenerative therapies (e.g., stem cell therapy [130], tissue engineering [131], gene therapy [132] and exogenous administration of growth factors [133]) aiming at increasing cardiomyocyte numbers and function have proven effective in pre-clinical trials and are currently undergoing clinical trials, whereby results to date have been ambiguous. 

## 5. Transthyretin Amyloidosis

Amyloidosis describes a heterogenous group of multi-systemic diseases caused by extracellular deposition of folded, insoluble and proteolysis resistant amyloid fibrils consisting of the precursor protein, proteoglycans and serum amyloid protein which results in alterations of tissue structure and consequently impairs organ function [134]. Currently, two types of systemic amyloidosis with relevant cardiac involvement have been identified: light-chain (AL) and transthyretin amyloidosis (ATTR) [135]. The first is considered to be the most prevalent form of amyloidosis and has therefore been extensively studied, the while latter has been considered rare. However, according to current scientific knowledge its prevalence and medical implications are far greater than assumed. In patients of African descent in the UK and USA it is estimated to be the fourth most common cause of congestive heart failure [136,137]. Furthermore, autopsy samples revealed a prevalence of transthyretin amyloidosis of 10%–25% in the elderly (age >80 years) [138], proving ATTR is clearly underdiagnosed [134]. Transthyretin (TTR) is an amyloidogenic protein primarily synthesized in the liver and secondarily (<5%) in the choroid plexus and retinal pigment epithelium and forms a tetramer loaded with transporting thyroxin and retinol-binding proteins [139,140]. Physiologically, a clinically negligible amount of TTR dissociates into dimers and monomers that subsequently polymerize into amyloid fibrils [141]. Point mutations resulting in changes in amino sequence and incompletely understood sporadic age-related mechanisms promote tetramer dissociation and misfolding and are the underlying causes for hereditary (hATTR or mATTR) and wild-type ATTR (wtATTR; formerly known as senile systemic amyloidosis). Characteristics off TTR types are shown in Table 2. Nearly 150 mutations exhibiting autosomal dominant inheritance with geographically and ethnically varying penetrance [142,143] have been identified in patients with hATTR. The most frequent single-nucleotide variant worldwide is the Val50Met mutation (formerly Val30Met before 20 positions were added), followed by the Val142Ile mutation endemic in the African American population (frequency 1:30) [137]. The largest clusters of Val50Met mutation exist in Portugal (prevalence 1:538 in northern regions), Sweden and Japan [134], whereas average prevalence in other populations (e.g., of northern/western European origin) is estimated far lower at 1:100,000 [144]. While hATTR typically presents itself in the third to fifth decade in endemic populations, wtATTR mainly effects the elderly and begins after age >70 years [139,145]. 

Depending on the underlying genotype, leading clinical syndromes of hATTR are familial amyloid cardiomyopathy (Val142Ile), familial amyloid polyneuropathy (Val50Met) and leptomeningeal amyloidosis (Asp3Gly) [156]. Clinical presentation of hATTR is highly heterogenous and often involves overlapping phenotypes instead of exclusive neuropathy or cardiomyopathy. Up to 43% of patients presenting with Val50Met and familial amyloid polyneuropathy (FAP) also exhibit cardiac amyloidosis which in turn is a frequent cause of death [139]. Likewise, in non-neuropathic forms of hATTR polyneuropathy may occur, but is often mild in manifestation. Wild-type ATTR on the other hand is associated with diffuse dispersion in numerous organs, whereby deposition is greatest in the heart. Accompanying illnesses of wtATTR are carpal tunnel syndrome, atraumatic rupture of the biceps tendon and lumbar canal stenosis [135,157]. Patients with wtATTR are predominantly male with rates between 72%–98% [158,159], albeit prevalence in women may be widely underestimated [160]. 

**Figure 1:**
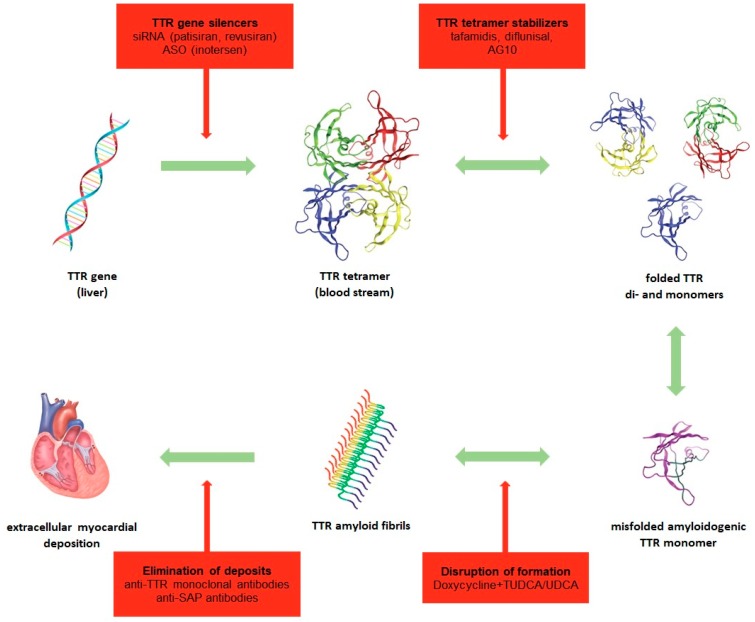
Pathogenetic therapy of TTR cardiac amyloidosis. TTR tetramers are prone to destabilization due to hereditary or wild point mutations in the *TTR* gene and dissociate into di- and monomers, which misfolds into an amyloidogenic form and aggregates to amyloid fibrils. TTR amyloid fibrils accumulate in the extracellular myocardium and induce cardiac dysfunction. Novel agents target singular points steps in the TTR amyloid cascade and thereby inhibit the development of TTR cardiomyopathy. siRNA (small-interfering RNA), ASO (anti-sense oligonucleotide), TUDCA/UDCA (tauroursodeoxycholic acid/ursodeoxycholic acid), SAP (SLAM-associated protein). Reprinted depictions of TTR tetramer, folded TTR di- and monomers and misfolded amyloidogenic monomer by permission from Proceedings of the National Academy of Sciences of the United States of America (Proc Natl Acad Sci USA. 2012 Jun 12;109:9629–9634: Bulawa CE, et al. Tafamidis, a potent and selective transthyretin kinetic stabilizer that inhibits the amyloid cascade.).

TTR cardiac amyloidosis is characterized by progressive infiltrative, restrictive cardiomyopathy with diastolic dysfunction (HFpEF) causing right-sided heart failure in early stages and deterioration of left systolic ejection fraction later on. The pathogenesis of TTR cardiac amyloidosis is shown in Figure 1. Additionally, infiltration of electrical pathways can result in conduction blocks and arrhythmias (e.g., atrial fibrillation). The diagnosis of ATTR requires multi-step diagnostic investigation comprising non-invasive and invasive techniques such as ECG, echocardiography, cardiac magnetic resonance imaging (CMRI) and cardiac scintigraphy as well as biomarkers, immunohistochemistry of biopsies and genetic testing. Unspecific pathologies in ECG common to cardiac amyloidosis are low voltage with poor R-wave progression and the pseudoinfarction pattern with prominent Q wave in leads II, III, aVF and V1-V3, presumably resulting from amyloid deposition in the anterobasal and -septal wall of the left ventricle [134,135,156]. The cornerstone of any diagnostic algorithm is echocardiography. Common signs of cardiac amyloidosis are left ventricular hypertrophy (LVH) with concentric or asymmetric pattern (cutoff >12 mm) [160,161], biventricular hypertrophy, diastolic dysfunction (high E/e ratio), left and biatrial dilatation, atrioventricular valve thickening, atrial septal wall thickening, reduced left ventricular ejection fraction, impaired longitudinal strain (especially in basal and midventricular segments, preserved in apical segments) and granular sparkling appearance of the myocardium [135,161,162]. However, in early stages of cardiac amyloidosis echocardiography may still be largely inconspicuous. Scintigraphy with ^99m^Tc-DPD shows cardiac tracer uptake and is capable of diagnosing TTR cardiac amyloidosis in early stages before echocardiographic or even CMRI abnormalities occur [163,164]. Other radiotracers such as ^99m^Tc-pyrophosphate, ^99m^Tc-hydroxymethylene and ^18^F-florbetapir have proven to be equally efficient [165,166,167]. Scintigraphy and CMRI can further differentiate between ATTR and AL with high sensitivity by determining tracer uptake or late gadolinium enhancement respectively and subsequently utilizing scoring systems [168,169]. CMRI might not be quite as sensitive and specific in diagnosing ATTR as nuclear imaging, yet sensitivity and specificity for cardiac amyloidosis is beyond 80% or 90% respectively [139]. Arising techniques such as quantitative T1 mapping (longer native T_1_ times) [135] and calculation of extracellular volume (higher in cardiac amyloidosis compared to other heart diseases) [170] may strengthen the role of CMRI in early diagnosis of ATTR. Furthermore, CMRI can distinguish between cardiac amyloidosis, hypertrophic cardiomyopathy and hypertensive heart disease which are all characterized by LVH in echocardiography. Late gadolinium enhancement over the entire subendocardial circumference is pathognomonic for cardiac amyloidosis, but transmural or patching patterns are also possible [171]. Important biomarkers for cardiac amyloidosis are NT-proBNP (N-terminal pro brain natriuretic peptide) and Troponin T or I, which are elevated due to direct toxicity of the TTR amyloid and myocardial strain. Especially, NT-proBNP appears to be a more sensitive marker due to significant correlation with left ventricular wall thickness and is therefore a useful prognostic marker in ATTR [172]. The two final steps for a definitive diagnosis are histological and genetic verification. The gold standard is an endomyocardial biopsy containing amyloid fibrils, whereby extracardiac biopsies (abdominal subcutaneous fat [173], bone marrow, rectal mucosa, nerves or minor salivary gland [174]) may provide a positive result when there is extracardiac deposition [138,175]. The latter is more frequent in hATTR than in wtATTR. A negative extracardiac biopsy though does not exclude a diagnosis of ATTR [135,139]. Thus, most patients with wtATTR will require an endomyocardial biopsy for confirmation. Leading techniques to differentiate between amyloid subtype are immunochemistry based on specific antibodies against amyloid proteins and mass spectrometry [176,177]. Finally, in order to distinguish hATTR from wtATTR, genetic sequencing must be conducted. In patients with hATTR it is further necessary to offer genetic counseling and follow-up on first-degree relatives, who may be asymptomatic carriers, as to commence clinical and diagnostic surveillance and therapy in early stages or even to prevent onset of disease [178,179]. 

Until recently, there was no approved causal therapy for ATTR apart from liver and/or heart transplantation. Even though orthotopic liver transplantation has proven successful in patients with FAP, it has been less effective in TTR cardiac amyloidosis with evidence of worsening cardiomyopathy due to post-implantation progressive deposition of native TTR [180,181,182]. Thus, the outcome of liver transplantation varies due to heterogeneity in mutations and patients’ overall medical status [183]. Therefore, targeted therapeutics to suppress synthesis of TTR (gene silencers), prevent tetramer dissociation (stabilizers) and eliminate depositions are currently being developed. At present, treatment of TTR cardiac amyloidosis mainly follows current guidelines for the management of heart failure and arrhythmias because research has primarily concentrated on studying the effects on FAP and less on ATTR cardiomyopathy. Therefore, existing pharmacological medications have so far only been approved for FAP. Patisiran (ALN-TTR02) is a double-stranded, small interfering RNA (siRNA) that has shown to reduce TTR production by >80% in hATTR and wtATTR [184]. In APOLLO, the largest randomized, double-blind, placebo-controlled, phase III study in patients with FAP treatment with patisiran significantly improved neurological symptoms and—as shown in a prespecified cardiac subpopulation (NYHA I and II)—was further associated with improvement in cardiac structure and function including significant reductions in left ventricular wall thickness, left ventricular longitudinal strain and NT-proBNP levels at 18 months [146,147]. Hence, patisiran was recently granted regulatory approval by the Food and Drug Administration (FDA) and the European Commission (EC) for the therapy of FAP. Revusiran (ALN-TTR01/ALN-TTRSC), a failed siRNA, was tested in patients with hATTR cardiomyopathy in the ENDEAVOUR phase III study that had to be discontinued due to sudden increase in mortality in the revusiran arm [148]. A further gene-silencing therapeutic agent that has passed a phase III clinical trial in patients with FAP (NEURO-TTR) is Inotersen (IONIS-TTR_Rx_), an antisense oligonucleotide (ASO). Results of NEURO-TTR showed a delayed progression of neurologic impairment, but no positive effect on cardiac status in a subpopulation with signs of cardiomyopathy at baseline [149]. However, a phase II trial undertaken by Benson et al. studying 22 patients with hATTR and wtATTR cardiomyopathy showed positive data regarding disease progression [150]. Marketing authorization for inotersen was approved from the EC for the treatment of stage 1 and 2 polyneuropathy in adults with hATTR, whereas regulatory approval was received from the FDA for FAP in adults. A phase III trial in patients with ATTR cardiomyopathy (CARDIO-TTR) was postponed due to severe thrombocytopenia and bleeding in the NEURO-TTR study. Continuation will depend on further data from ongoing trials. The first pharmaceutical expected to be approved for treatment of ATTR cardiomyopathy is tafamidis, a TTR tetramer stabilizer, that while being less effective in FAP [185,186] delivered promising results in the phase III trial ATTR-ACT [151] studying patients with ATTR cardiomyopathy over 30 months. Compared to the placebo, tafamidis reduced all-cause mortality and frequency of cardiovascular events in patients with hATTR and wtATTR amyloidosis. Furthermore, 6MWT and quality of life were significantly improved, while NT-proBNP levels and echocardiographic parameters showed positive trends. An extension phase III trial with treatment for up to 60 months vs. placebo has been approved and will end in 2021. Diflunisal, a nonsteroidal anti-inflammatory agent that stabilizes the TTR tetramer, has not yet been associated with a relevant effect on ATTR cardiomyopathy [152,153]. Moreover, diflunisal negatively affected kidney and gastrointestinal function causing water retention and hypertension, adverse effects that are counterproductive in heart failure. Therefore, use of diflunisal remains limited to off-label use in treatment of FAP. A number of novel TTR stabilizers with higher potency for tetramer stabilization such as AG-10 [187], CSP-1103 and SOM0226 are currently in development for ATTR cardiomyopathy. Likewise, progress is being made regarding agents targeting the elimination of TTR amyloid deposits. Current focus is on the combination of doxycycline, an antibiotic that disrupts the formation of amyloid fibrils and tauroursodeoxycholic acid (TUDCA), a biliary acid that reduces non-fibrillar TTR aggregates. Results from phase II studies have been inconsistent with some suggesting a protective effect with delay in progression of ATTR cardiomyopathy, while others negate said findings [154,155]. Hence, further research is necessary in order to draw a definite conclusion. Similarly, research on other deposit eliminating agents such as anti-TTR monoclonal antibodies (e.g., PRX004) that promote phagocytosis of TTR amyloid aggregates and anti-SAP antibodies (e.g., dezamizumab) is in its early stages [188,189]. Regardless of the substance group future studies must concentrate on not only proving effectiveness in ATTR cardiomyopathy, but also distinguish which subgroups benefit the most (tafamidis for example showed response to therapy in NYHA class I and II, but not in NYHA class III [151]) and whether combinations of drug groups (e.g., dual therapy with gene-silencer and TTR stabilizer) are more potent in improving cardiac structure and function than monotherapy. Selected phase II and III studies for treatment of TTR amyloidosis are presented in Table 3.

## 6. Conclusions

HF is a heterogenous syndrome with diverse etiologies, pathological mechanisms and clinical presentations. In view of the fact that ischemic HF and HFpEF constitute the absolute majority of HF cases, much effort has gone into investigating their underlying pathophysiology. Despite an already extensive body of pre-clinical research immunological pathways in both forms of HF are far from understood. Cardiac inflammation following ischemia and mechanical stress encompasses an intricate and complex interplay between immune cells and various pro- and anti-inflammatory mediators responsible for removal of damaged tissue and reparation of cardiac tissue, thereby preventing fatal rupture and upholding cardiac function. Some of the immune mediators participating in this process cannot be simplified into being solely pro- or anti-inflammatory, but are dichotomous depending on inflammatory phase and environment. The emergence and progression of HF results from excessive and sustained inflammation, which in part is facilitated by autoimmunity through the adaptive immune system. In order to develop effective treatment options, specific immunological mechanisms must be further characterized and studied in patients with acute and chronic HF, so that therapeutic drugs can be administered in the right populations at the appropriate time point. Furthermore, it is essential to acknowledge that HF cannot be managed successfully with a uniform treatment but requires individualized strategies that also look beyond the heart and towards comorbidities. This is underscored by the differences in pathophysiology of ischemic HF and HFpEF. With that in mind, other forms of HF that may be concurrent should not be neglected. Until recently, TTR cardiac amyloidosis was disregarded due to the assumption it was rare. New data on the prevalence of ATTR cardiomyopathy as an independent disease but also as a concomitant pathology in HF of other primary origin has garnered attention and paved the way for intensified research, which has culminated in the development of refined diagnostic algorithms and effective targeted therapies.

## Figures and Tables

**Table 1:** Selected clinical trials targeting immunosuppressive and immunomodulatory therapies in myocardial infarction and heart failure.

Immunosuppression					
Trial	Study Population	*n*	Treatment	Follow-Up	Primary Outcome
**Corticosteroids**					
COPE-ADHF [88]	Acute decompensated HF	102	Dexamethasone or prednisolone 20 mg IV once daily; 1 mg/kg daily (max. 60 mg) for 7 days then tapered off vs. standard treatment	19 months	Reduced cardiac mortality, improvement of dyspnea and global clinical status
Mentzelopoulos et al. [89]	Cardiac arrest	268	Methylprednisolone 40 mg IV once and hydrocortisone 300 mg IV daily for 7 days then tapered off vs. saline placebo	2 months	Improved rate of ROSC, survival to discharge and neurological outcome
Tsai et al. [90]	Cardiac arrest	97	Hydrocortisone 100 mg IV during resuscitation vs. saline placebo	7 days	Higher ROSC rate, no difference in survival and hospitality discharge rates
**Methotrexate**					
TETHYS [91]	STEMI	84	0.05 mg/kg + 0.05 mg/kg/h for 6 days vs. placebo	3 months	No difference in mortality, coronary blood flow, infarct size, cardiac markers or reinfarction, worsened LVEF at 3 months
CIRT [92]	Prior MI and either type 2 diabetes or metabolic syndrome	7000	Target dose 15–20 mg/week	3–5 years	Results pending
METIS [93]	Ischemic congestive HF	50	7.5 mg/week for 12 weeks	3 months	Tendency toward improved NYHA, no difference in 6MWT or MACE
**Cyclosporine A**					
Yingzhong et al. [94] (meta-analysis): CYCLE (2016) Cung et al. (2015) Ghaffari et al. (2013) Mewton et al. (2010) Piot et al. (2008)	Acute MI / STEMI	∑ 1250	2.5 mg/kg IV vs. placebo	6 months, Cung 12 months	No difference in all-cause mortality or adverse clinical events, no significant improvement of LVEF or infarct size
**IVIg**					
Gullestad et al. [95]	Acute MI treated by PCI	62	0.4 g/kg once daily for 5 days then 0.4 g/kg monthly for 26 weeks vs. placebo	6 months	No effect on LV remodeling or function and inflammatory markers after completed maintenance therapy
Gullestad et al. [96]	Congestive HF and LVEF <40%	40	0.4 g/kg once daily for 5 days then 0.4 g/kg monthly for 5 months vs. placebo	6 months	Marked rise of anti-inflammatory markers and significant increase in LVEF
IMAC [97]	Recent onset of idiopathic DCM and LVEF <40%	62	1 g/kg IVIG for 2 days vs. placebo	12 months	No difference in LVEF improvement
**Immunomodulation**					
**IL-1 inhibitors**					
DHART 2 [98]	HFpEF and CRP >2 mg/L	31	Anakinra 100 mg sc daily vs. placebo for 12 weeks vs. placebo	24 weeks	No improvement in cardiorespiratory fitness
Van Tassell et al. [99]	Acute decompensated HF, LVEF <40% and CRP ≥5 mg/L	30	Anakinra 100 mg sc twice daily for 3 days followed by once daily for 11 days vs. placebo	14 days	Reduction in systemic inflammatory response, no evaluation of cardiac function/clinical outcomes
MRC-ILA Heart Study [100]	NSTEMI <48 h from onset of chest pain	182	Anakinra 100 mg sc for 14 days vs. placebo	12 months	Reduction in inflammatory markers, higher rate of MACE at 1 year
VCU-ART [101]	Acute MI	10	Anakinra 100 mg sc daily for 14 days	14 weeks	Favorably affected LV end-systolic and -diastolic volume index
Everett et al. [102] Ridker et al. [103] (CANTOS)	Prior MI and high-sensitivity CRP ≥2 mg/L	10,061	Canakinumab 50, 150, or 300 mg sc once every 3 months vs. placebo	3.7 years	Dose-dependent reduction in hospitalization for HF and the composite of hospitalization or HF-related mortality, lower rate of recurrent cardiovascular events
Trankle et al. [104] (CANTOS sub study)	Prior MI, high-sensitivity CRP ≥2 mg/L and LVEF <50%	30	Canakinumab 50, 150 or 300 mg sc once every 3 months vs. placebo	12 months	Improvement of cardiorespiratory fitness and LVEF
**IL-6 receptor antagonist**					
Kleveland et al. [105]	NSTEMI	117	Tocilizumab 280 mg IV single dose vs. placebo prior to coronary angiography	6 months	Attenuation of inflammatory response (hs-CRP, leukocytes, hs-TNT)
**TNF-α inhibitors**					
RENEWAL [106] (RECOVER and RENAISSANCE)	Chronic HF, NYHA II-IV and LVEF ≤30%	925 + 1123	Etanercept 25 mg sc once or twice a week vs. placebo;etanercept 25 mg sc twice or three times per week vs. placebo	24 weeks	No effect on clinical status, hospitalization due to chronic HF or mortality
ATTACH [107]	Chronic HF, NYHA III-IV and LVEF ≤35%	150	Infliximab 5 or 10 mg/kg or placebo at 0, 2 and 6 weeks	28 weeks	No improvement after short-term treatment, higher risk of hospitalization due to HF and death under 10 mg/kg
**Complement inhibitors**					
Fattouch et al. [108] (C1 esterase inhibitor)	STEMI undergoing emergent CABG	80	C1-INH 1000 UI vs. placebo	48 h	Improved cardiac function (CI, SV) and haemodynamics without impact on early mortality rate
Testa et al. [109] (meta-analysis of C5-inhibitor, 6 studies)	STEMI or elective CABG	∑ 15,915	Pexelizumab 2 mg/kg + 0.05 mg/kg/h for 24 days; pexelizumab 2 mg/kg or 2 mg/kg + 0.05 mg/kg/h for 20 days	7 days, 3 months, 6 months	In STEMI no benefit in MACE, MI, stroke or heart failure; in CABG 26% reduction in risk of death
**Targeting ROS and NO-cGMP-PKG signaling**					
NACIAM [110]	STEMI	112	High-dose N-acetylcysteine (29 g over 2 days) with background low-dose nitroglycerin (7.2 mg over 2 days) vs. placebo	3 months	Increased myocardial salvage and reduced infarct size, clinical outcomes not assessed
SOCRATES-PRESERVED [111]	HFpEF (LVEF ≥45%)	477	Vericiguat once daily at 1.25 or 2.5 mg fixed doses, or 5 or 10 mg titrated from a 2.5 mg starting dose, or placebo for 12 weeks	12 weeks	No change in NT-proBNP or left atrial volume, improvement in quality of life
**Targeting adaptive immunity**					
Gao et al. [112] (meta-analysis)	Acute MI	1736	Adenosine in varying doses		No improvement of LVEF, all-cause mortality, cardiovascular mortality or re-infarction after PCI
PRESTO [113] (mast cell stabilizer)	PCI of at least one vessel stenosis	11,484	Tranilast 300 mg or 450 mg twice daily oral for 1 month or 3 months	9 months	No improvement of mortality, MACE or target vessel revascularization
Kim et al. [114] (histamine H2 receptor antagonist)	Symptomatic congestive HF	50	Famotidine 30 mg daily for 6 months vs. teprenone	6 months	Improved both cardiac symptoms, ventricular remodeling (LVEDV/LVESV) and MACE
HALT-MI [115] (CD11/CD18 integrin inhibitor)	STEMI within 6 h of onset of chest pain	420	Hu23F2G (Leukoarrest) 0.3 or 1 mg/kg IV bolus or placebo	1 month	No difference in infarct size, mortality or MACE

6MWT, 6-min walk test; C1-INH, C1-inhibitor; CABG, coronary artery bypass graft; CI, cardiac index; DCM, dilated cardiomyopathy; HF, heart failure; HFpEF, heart failure with preserved ejection fraction; hs-CRP, high-sensitivity C-reactive protein; hs-TnT, high-sensitivity troponin T; IV, intravenous; IVIg, intravenous immunoglobulin; LV, left ventricular; LVEDV, left ventricular end diastolic volume; LVEF, left ventricular ejection fraction; LVESV, left ventricular end systolic volume; MACE, major adverse cardiac event; MI, myocardial infarction; NSTEMI, non-ST elevation myocardial infarction; NYHA, New York Heart Association; PCI, percutaneous coronary intervention; ROS, reactive oxygen species; ROSC, return of spontaneous circulation; sc, subcutaneous; STEMI, ST-segment elevation myocardial infarction; SV, stroke volume;.

**Table 2:** Characteristics of wild-type transthyretin amyloidosis (wtATTR) and hereditary transthyretin amyloidosis (hATTR).

	wtATTR	hATTR
Prevalence	Unknown prevalence, higher than thus far assumed, probably very frequent and perhaps leading form of amyloidosis	<1:100,000
Pathogenesis	Sporadic misfolding	Point mutations, most frequent: Val50Met (~73%) and Val142Ile (~4%)
Age	>60 years, especially in elderly >80 years, rarely diagnosed during life	At younger age <60 years (30–50 years), depending on mutation
Sex	Male predominance	Male predominance with more aggressive phenotype
Clinical course	Often asymptomatic	Dependent on mutation and penetrance homozygosity linked to higher incidence, earlier onset and more severe clinical presentation strong genotype-phenotype correlation
Affected organs	Dispersed deposition in several organs: primarily cardiac deposition and secondarily neural deposition; eye, kidney and tendon involvement also possible	Val50Met: polyneuropathy, in 43% also cardiac involvement Val142Ile: cardiomyopathy, in 30% also polyneuropathy other mutations with leptomeningeal, ophthalmological and nephrological involvement
Cardiac injury	Progressive cardiomyopathy with hypertrophy, diastolic (early) and systolic dysfunction (late) Conduction disorders Atrial arrhythmias, e.g., fibrillation Degenerative aortic stenosis	
Extracardiac injury	Carpal tunnel syndrome Lumbar spinal stenosis Atraumatic biceps tendon rupture	Polyneuropathy:ascending bilateral sensory-motor polyneuropathy; dysautonomia (e.g., orthostatic hypotension, gastrointestinal, erectile dysfunction) Eye disease: glaucoma, intravitreal deposition, scalloped pupils Nephropathy: nephritic syndrome, progressive renal failure
Diagnostic methods for cardiomyopathy	ECG, echocardiography, cardiac MRI, cardiac scintigraphy	

**Table 3:** Selected phase II/III trials for TTR amyloidosis.

Substance Group	Agent	Trial and Design	Investigated Population	Efficacy Endpoints Regarding Cardiac Status	Pending Approvals/Trials in Planning
*TTR* gene silencer	Patisiran	**APOLLO** Phase III [146,147] Randomized, double-blind, placebo-controlled	225 patients with FAP; 56% with cardiac involvement (subgroup analysis) Randomized 2:1 0.3 mg/kg patisiran IV or placebo every 3 weeks for 2 years Follow-up 18 months	- Reduced left ventricular wall thickness - Increased end-diastolic volume - Decreased global longitudinal strain - Increased cardiac output - Lowered NT-proBNP	Regulatory approval granted from FAD and EC for the therapy of FAP Vutrisiran vs. patisiran in hATTR (**HELIOS-A**; currently recruiting)
	Revusiran	**ENDEAVOUR** Phase III [148] Randomized, double-blind, placebo-controlled	206 patients with FAC Revusiran 500 mg SC for 5 days, then weekly for 18 months years vs. placebo		Discontinued due to increase in mortality in the revusiran arm
	Inotersen	**NEURO-TTR** Phase III [149] Randomized, double-blind, placebo-controlled	172 patients with FAP stage I and II Randomized 2:1 300 mg SC every 12 h for 1 week, then weekly for 64 weeks vs. placebo Follow-up 15 months	- No improvement in structure or function in subgroup with cardiac involvement at baseline	Marketing authorization approved from EC for treatment of stage 1+2 PNP in hATTR; regulatory approval from the FDA for FAP
		**Phase II study** [150] open-label, non-randomized	20 patients with ATTR cardiomyopathy Inotersen 300 mg SC every 12 h for 1 week	- Stable cardiac disease: no increase in strain, reduction of LV mass	
		**CARDIO-TTR**	Trial in patients with FAC		Postponed due to increased thrombocytopenia and bleeding in NEURO-TTR
TTR stabilizer	Tafamidis	**ATTR-ACT** Phase III [151] Randomized, double-blind, placebo-controlled	441 patients with Randomization 2:1:2 tafamidis 80 or 20 mg or placebo orally every 24 h for 30 months	- Decrease in all-cause mortality and cv rehospitalization - Delayed decline in distance for 6-min walk test - Delayed decline in KCCQ-OS score - Positive trends in NT-proBNP levels and echocardiographic parameters	Extension phase up to 60 months vs. placebo Approval from EC for use in FAP stage I 01/2019 FDA accepts regulatory submissions for review to treat TTR cardiomyopathy
	Diflunisal	**Phase III study** [152]	130 patients with FAP 50% with cardiac involvement at baseline Randomization 1:1 Diflunisal 250 mg orally every 12 h vs. placebo for 24 months	- No improvement in cardiac status - Negative effect on kidney and gastrointestinal function	Off-label use in FAP Further efficacy trials required
		**Phase II study** [153] Single-arm, open-label	13 patients with ATTR cardiomyopathy	- No significant change in cardiac structure or function or in biomarker levels	
	AG-10	**Phase II study** Randomized, double-blind, placebo-controlled (NCT03458130)	45 patients with ATTR cardiomyopathy (at least 30% hATTR) Randomization 1:1:1 two different doses of AG10 every 12 h or placebo	- Study ended in March 2018, results pending	Further efficacy trials expected (e.g., NCT03536767)
Elimination of deposits	Doxycycline + TUDCA/UDCA	**Phase II study** [154]	53 patients with ATTR cardiomyopathy treated with doxycycline and ursodiol, follow-up 22 months	- Stabilized cardiac biomarker - Improved global longitudinal strain in less advanced disease	Further efficacy trials pending
		**Phase II study** [155]	55 patients in patients with ATTR cardiomyopathy Doxycycline 100 mg orally every 12 h for 4 weeks with a pause of 2 weeks, then UDCA 750 mg (500 + 250 mg) orally per day continuously for 12 months	- No changes in NT-proBNP at 6 months, increase at 12 months - Stable LVH - High dropout rate	
	Anti-SAP	**Phase II study** Open-label, non-randomized, three groups (NCT03044353)	40 patients Cohort 1 with ATTR cardiomyopathy Cohort 2 AL at >6 months post chemotherapy Cohort 3 newly diagnosed AL Anti-SAP+CPHPC monthly for 6 months; follow up max. 18 months	- Suspended pending data review	

## Abbreviations

6MWT	6-min walk test
99mTc-DPD	Tc-99m-3,3-diphosphono-1,2-propanodicarboxylicacid
AL	light chain amyloidosis
APC	antigen presenting cells
ASO	anti-sense oligonucleotide
ATII	angiotensin II
ATTR	transthyretin amyloidosis
CCL2	CC-chemokine ligand 2
CCR2	C-C chemokine receptor type 2
cGMP	cyclic guanosine monophosphate
CMRI	cardiac magnetic resonance imaging
CRP	C-reactive protein
CsA	cyclosporin A
DAMPs	danger-associated molecular patterns
DCs	dendritic cells
EC	European Commission
ECG	electrocardiogram
ECM	extracellular matrix
FAP	familial amyloid polyneuropathy
FDA	Food and Drug Administration
hATTR	hereditary transthyretin amyloidosis
HF	heart failure
HFpEF	heart failure with preserved ejection fraction
HFrEF	heart failure with reduced ejection fraction
ICAM1	intracellular adhesion molecule 1
IVIg	immunoglobulins
IL-1	Interleukin-1
IL-1β	Interleukin-1β
IL-10	Interleukin-10
IL-6	Interleukin-6
LVEF	left ventricular ejection fraction
LVH	left ventricular hypertrophy
LYVE-1	lymphatic vessel endothelial hyaluronan receptor 1
MI	myocardial infarction
MMPs	matrix metalloproteinases
MRI	magnetic resonance imaging
MTX	methotrexate
NAC	N-acetylcysteine
NF-κB	nuclear factor kappa-light-chain-enhancer of activated B-cells
NO	nitric oxide
NT-proBNP	N-terminal prohormone of brain natriuretic peptide
PCI	percutaneous coronary intervention
PKG	protein kinase G
ROS	reactive oxygen species
SAP	SLAM-associated protein
sGC	soluble guanylyl cyclase
siRNA	small-interfering RNA
STEMI	ST-elevated myocardial infarction
TAC	transverse aortic constriction
TGF-β	transforming growth factor beta
TNF-α	Tumor necrosis factor alpha
TNFR1	TNF receptor 1
TNFR2	TNF receptor 2
TTR	transthyretin
TUDCA	tauroursodeoxycholic acid
UDCA	ursodeoxycholic acid
VEGF	vascular endothelial growth factor
VEGF-C	vascular endothelial growth factor-C
VCAM1	vascular cell adhesion protein 1
wtATTR	wild-type transthyretin amyloidosis
